# Recombinant L-asparaginase 1 from *Saccharomyces cerevisiae*: an allosteric enzyme with antineoplastic activity

**DOI:** 10.1038/srep36239

**Published:** 2016-11-08

**Authors:** Iris Munhoz Costa, Leonardo Schultz, Beatriz de Araujo Bianchi Pedra, Mariana Silva Moreira Leite, Sandra H. P. Farsky, Marcos Antonio de Oliveira, Adalberto Pessoa, Gisele Monteiro

**Affiliations:** 1 Department of Biochemical and Pharmaceutical Technology, School of Pharmaceutical Sciences, University of São Paulo, São Paulo/SP 05508-000, Brazil; 2 Biosciences Institute, São Paulo State University - UNESP, Coastal Campus, São Vicente/SP 11330-900, Brazil; 3 Department of Clinical and Toxicological Analysis School of Pharmaceutical Sciences, University of São Paulo, São Paulo/SP 05508-000, Brazil

## Abstract

L-asparaginase (L-ASNase) (EC 3.5.1.1) is an important enzyme for the treatment of acute lymphoblastic leukaemia. Currently, the enzyme is obtained from bacteria, *Escherichia coli* and *Erwinia chrysanthemi*. The bacterial enzymes family is subdivided in type I and type II; nevertheless, only type II have been employed in therapeutic proceedings. However, bacterial enzymes are susceptible to induce immune responses, leading to a high incidence of adverse effects compromising the effectiveness of the treatment. Therefore, alternative sources of L-ASNase may be useful to reduce toxicity and enhance efficacy. The yeast *Saccharomyces cerevisiae* has the *ASP1* gene responsible for encoding L-asparaginase 1 (ScASNase1), an enzyme predicted as type II, like bacterial therapeutic isoforms, but it has been poorly studied. Here we characterised ScASNase1 using a recombinant enzyme purified by affinity chromatography. ScASNase1 has specific activity of 196.2 U/mg and allosteric behaviour, like type I enzymes, but with a low *K*_*0.5*_ = 75 μM like therapeutic type II. We showed through site-directed mutagenesis that the T64-Y78-T141-K215 residues are involved in catalysis. Furthermore, ScASNase1 showed cytotoxicity for the MOLT-4 leukemic cell lineage. Our data show that ScASNase1 has characteristics described for the two subfamilies of l-asparaginase, types I and II, and may have promising antineoplastic properties.

L-asparaginase amidohydrolase (L-ASNase) (EC 3.5.1.1) catalyses the hydrolysis of L-asparagine (L-Asn) in aspartic acid and ammonia. L-ASNases are classified in three families: the plant-type, the *Rhizobium etili*-type and the bacteria-type. The bacterial family is subdivided into type I and type II^1^; type I enzymes are cytosolic, expressed constitutively and have low affinity for L-Asn, resulting in non-therapeutic applications, while type II enzymes are restricted in the periplasmic space, with induced expression during anaerobiosis, have high affinity for L-Asn and present antitumor activity^2^,^3^.

L-ASNase type II from *Escherichia coli* (EcASNase2) and *Erwinia chrysanthemi* (EwASNase2), native and PEGylated forms, are used for the treatment of acute lymphoblastic leukaemia (ALL) due to the fact that leukemic cells need extracellular L-Asn for protein synthesis, and L-ASNase depletes L-Asn and L-glutamine (L-Gln) from serum causing death by starvation and the absence of anti-apoptotic proteins^4^,^5^,^6^. Interestingly, only leukaemia cells are sensitive to L-ASNase, as they frequently present asparagine synthetase (ASNS) genes that have been silenced by epigenetic mechanisms, while normal blood cells do not^7^,^8^.

However, during treatment with bacterial L-ASNases, patients show a high incidence of adverse effects, such as neurotoxicity caused by the hydrolysis of L-Gln, hypersensitivity and allergic reactions that can lead to anaphylactic shock, and the formation of antibody anti-asparaginase^4^,^9^,^10^,^11^,^12^. Nowadays, new L-ASNases have been identified in eukaryotic sources, in an effort to find new promising biopharmaceuticals with fewer side effects^13^.

The yeast *Saccharomyces cerevisiae* constitutively expresses the gene *ASP1,* producing an intracellular cytoplasmic enzyme L-asparaginase 1 (ScASNase1). However, it has high molecular similarity to bacterial enzymes used in therapy^14^,^15^ and is classified in the bacterial type II family^1^,^16^. In the 1970s, the few studies performed with ScASNase1 demonstrated low activity and affinity to L-Asn^14^,^17^. Since then, no studies about this enzyme have been reported.

Therefore, we here describe the structural and biochemical characterization of the recombinant ScASNase1. Our results revealed that ScASNase1 presents allosteric behaviour similar to that of type I enzymes. Using site-directed mutagenesis approach to substitute important residues used in catalysis in bacterial enzymes, which are conserved in ScASNase1, we show that substitutions abrogated the enzyme activity but do not exert significant effects on the secondary structure of the enzyme. Moreover, we have demonstrated that ScASNase1 has antineoplastic potential in the MOLT-4 leukemic cell lineage, similarly to that observed for type II bacterial enzymes.

## Results

### Determining specific activity, optimum pH, temperature and kinetic parameters of ScASNase1

The *ASP1* gene from *S. cerevisiae* has 1,146 bp and was obtained by PCR from genomic DNA, then cloned into the expression vector pET15b. The ScASNase1 was expressed in cytosol in the soluble fraction of proteins from the *E. coli* BL21 (DE3) strain. After purification, the enzyme was analysed by SDS–PAGE. The molecular mass of ScASNase1 is 41.4 kDa, and the His-tag addition resulted in a recombinant enzyme of approximately 45 kDa (http://web.expasy.org/protparam/). The gel showed a homogenous and pure protein with the expected molecular weight (see supplementary Fig. S1).

**Figure 1:**
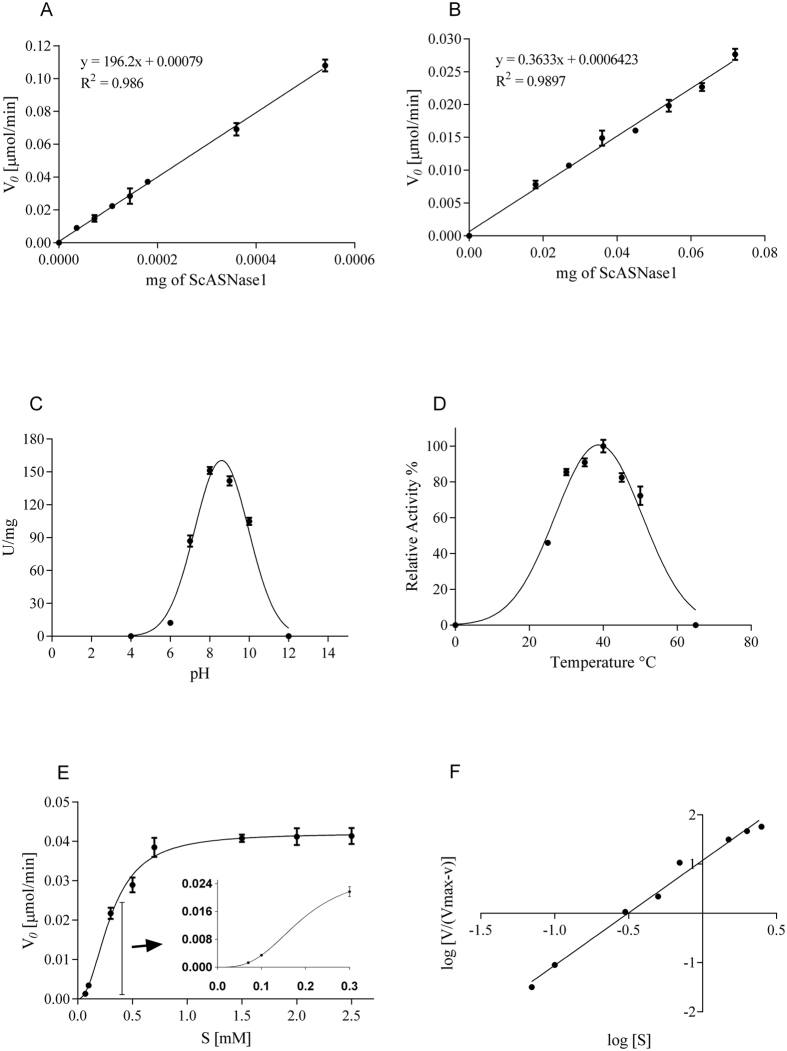
Determination of specific activity, optimum reaction conditions and kinetic characterisation for ScASNase1 enzyme. (**A**) Specific activity of ScASNase1 to hydrolyse L-Asn: Plot of the reaction velocities (*V*_*0*_) of L-Asn hydrolysis as a function of mg of purified ScASNase1 as measured by Nessler’s reagent. (**B**) Specific activity of ScASNase1 for L-Gln as measured by coupled assay with NADH oxidation: Plot of the reaction velocities (*V*_*0*_) of L-Gln hydrolysis as a function of mg of purified ScASNase1. (**C**) The effect of pH was determined in different buffers (acetate pH 4.0; sodium phosphate pH 6.0, 7.0 and 12.0; Tris–HCl pH 8.0 and 9.0; sodium bicarbonate pH 10.0). (**D**) Optimum temperature was determined by measuring the specific activity in the range from 25 °C to 50 °C. (**E**) ScASNase1 kinetics, activity dependence on a substrate concentration plot. The inset shows the sigmoidal profile of the enzyme at lower substrate concentrations. (**F**) Hill plot of the data. Points in the graph represent means ± SD (n = 3).

Using the purified protein, we determined the specific activity for L-Asn with Nessler’s reagent and by coupled assay with NADH oxidation for L-Gln. The specific activity was calculated by the initial velocity of L-Asn or L-Gln hydrolysis as a function of enzyme concentration (Fig. 1A,B). ScASNase1 presented high specific activity for L-Asn of 196.2 ± 5.8 U/mg and low for L-Gln of 0.4 ± 0.02 U/mg; this last represents 0.38% of the L-asparaginase activity, considering the determination of the activity by the same method for both substrates (see supplementary Fig. S2). The pH effect on the optimum activity of the enzyme was measured in the range from 4.0 to 12.0. ScASNase1 was active in the range from pH 5.0 to 11.0, and its optimal activity was at pH 8.6 (Fig. 1C). The optimum temperature was determined by measuring enzyme activity in a temperature range from 20 °C to 65 °C for 20 minutes, and the higher value observed was at 40 °C (Fig. 1D).

The kinetic parameters were determined by coupled assay with NADH oxidation. Different concentrations of L-Asn were used to create a plot of initial velocity as a function of L-Asn concentration; the initial velocity was determined through linear regression. The graph revealed a sigmoidal profile and allosteric behaviour (Fig. 1E) and affinity for the substrate in the μM range with positive cooperativity, as indicated by the Hill plot (Fig. 1F). The Hill coefficient measures the deviation of the Michaelis–Menten kinetics and describes quantitatively the degree of enzyme cooperativity^18^. The kinetic parameters determined are presented in Table 1. Statistical analysis of the kinetic models (Michaelis–Menten and sigmoidal allosteric), using the F-test under the null hypothesis that the enzyme has a Michaelis–Menten kinetic profile, revealed that the best kinetic model for ScASNase1 is the allosteric sigmoidal model with R^2^ = 0.9726 and p < 0.001. The commercial EcASNase2 enzyme used as the control showed michaelian behaviour with R^2^ = 0.9797 and *K*_m_ in the μM range (Table 1), in agreement with the described in the literature, and specific activity of 224 ± 7 U/mg (see supplementary Fig. S3).

**Figure 2:**
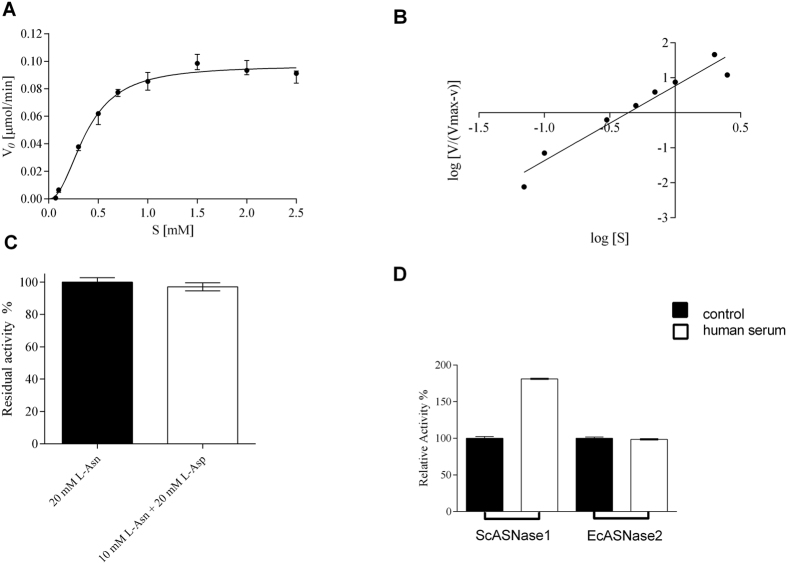
Influence of L-Asp and L-Gln on the parameters of ScASNase1. (**A**) ScASNase1 kinetic parameters using L-Asn and L-Gln together as substrates at the ratio of 1:16; activity dependence on a substrate concentration plot. (**B**) Hill plot of the data. Points represent means ± SD (n = 3). (**C**) Specific activity of ScASNase1 with added L-aspartate. 20 mM L-asparagine (L-Asn, 20 mM) and 10 mM L-asparagine + 20 mM L-aspartate (20 mM L-Asn + 10 mM L-Asp). (**D**) Specific activity of ScASNase1 and EcASNase2 after incubation at 37 °C for 60 minutes with 10% human serum. The graphs of specific activities and kinetic parameters were obtained with GraphPad Prism 6.05 software. The specific activity was quantified by Nessler’s reagent. Error bars were calculated from the mean ± SD (n = 3).

EcASNase2 has recently been described as possessing profile with allosteric positive cooperativity (n_H_ = 1.5) regulated by L-Gln^19^. At submicromolar concentrations of L-Asn, as occurs in the blood for the treatment of ALL, L-Gln is the most abundant substrate competing for the active site, and so the enzyme begins to present n_H_ = 1.0, which features Michaelis–Menten kinetics. ScASNase1 showed allosteric behaviour using only L-Asn as the substrate. To evaluate whether L-Gln could regulate the degree of cooperativity for ScASNase1, we also determined the kinetic parameters in the presence of L-Asn and L-Gln at a ratio of 1: 16 for each concentration of L-Asn. However, the addition of L-Gln did not alter the kinetic profile of the enzyme, different of observed to EcASNase2^19^ (Fig. 2A,B, Table 1).

Moreover, allosteric enzymes have the characteristic of being inhibited by the product of the reaction^20^,^21^; the specific activity of ScASNase1 in the presence of 20 mM L-aspartate showed that the product is not able to interfere with enzyme activity (Fig. 2C). We also tested whether certain ions can act as enzyme–cofactors. The addition of Zn^2+^ and Ca^2+^ resulted in the decrease of ScASNase1 activity. No difference was detected when Mg^2+^ was used, whereas K^+^ increased the level of enzymatic activity (Table 2). Since Zn^2+^ and Ca^2+^ diminished the activity of ScASNase1 *in vitro*, we evaluated the effect of the enzymes incubation with human serum over the specific activity of ScASNase1 and EcASNase2; we observed an increase in the activity and no effect, respectively (Fig. 2D), suggesting that in human serum ScASNase1 can be up stimulated and not repressed.

### Site-directed mutagenesis of ScASNase1

**Figure 3:**
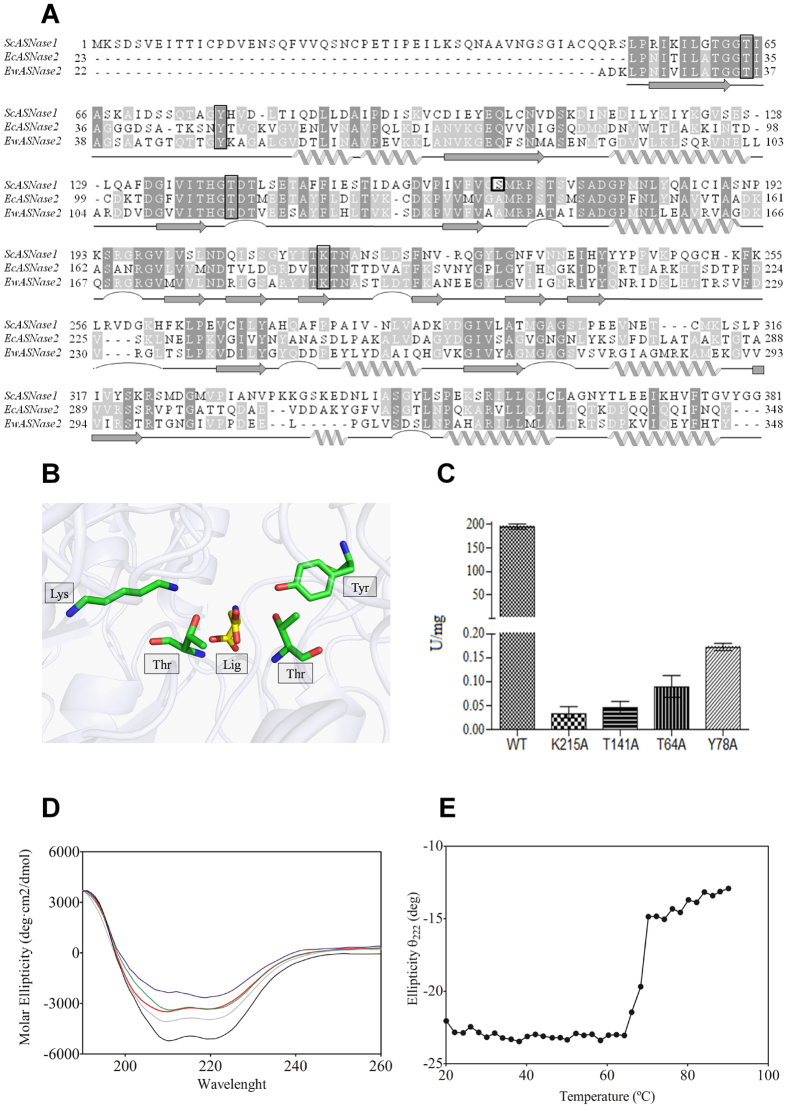
Structural and mutational analysis of ScASNase1. (**A**) Alignment of L-asparaginase amino acid sequences of *S. cerevisiae* (ScASNase1) (Uniprot number P38986), *E. coli* (EcASNase2) (Uniprot number P00805) and *E. chrysanthemi* (EwASNase2) (Uniprot number P06608). The amino acids are shaded in grey tones according to physico-chemical similarities among the three isoforms. The black boxes denote conserved amino acids involved in catalytic activity. The alignment was produced using Clustal Ω (http://www.ebi.ac.uk/Tools/msa/clustalo/), and the graphical representation was generated using JalView (http://www.jalview.org). (**B**) Crystallographic structure of *E. coli* (PDB code = 3ECA), highlighting the position of conserved residues and a ligand molecule. (**C)** Specific activity of the active site residues mutated in ScASNase1 by an alanine. The error bar represents means ± SD (n = 3). (**D**) Circular dichroism spectra of the ScASNase1 (black) and the mutant enzymes T64A (green), Y78A (red), K125A (grey) and T141A (blue). The protein concentrations used in experiments were 5 μM in Tris buffer 10 mM (pH 7.4). All spectra were recorded at 25 °C and corrected against the buffer. The graphical representations are averages from eight consecutive scans. (**E**) The thermal melting profile for ScASNase1 was recorded at 222 nm, and the temperature was increased from 20 to 90 °C. The protein concentrations used in experiments were 5 μM in Tris buffer 10 mM (pH 7.4). The melting temperature obtained was 68.25 ± 0.21 °C.

ScASNase1 shares 38% sequence identity with bacterial type II counterparts, and amino acids already determined as essential to enzyme catalysis are strictly conserved between bacterial enzymes and ScASNase1 (Fig. 3A,B). To verify the importance of conserved catalytic residues to the mechanism of ScASNase1, four residues were replaced individually with alanine by site-directed mutagenesis (highlighted in Fig. 3A). The mutants T64A, Y78A, T141A and K215A were expressed intracellularly and soluble in the BL21 (DE3) *E. coli* strain. The isoforms were purified and analysed by SDS–PAGE. The molecular mass of the isoforms was approximately 45 kDa with the His-tag, and the enzymes obtained were homogeneous and pure (see supplementary Fig. S4). The activities of isoforms were measured by Nessler’s reagent in the same conditions as the wild-type enzyme, and a 99.9% loss of activity was observed for all mutants (Fig. 3C). Statistical analysis of the specific activities of the T64A, K215A and T141A mutants showed no significant difference between them. The Y78A mutant presented a significant difference when compared to others, p ≤ 0.05.

Recently, similar mutations were performed to the L-asparaginase from guinea pig AspG (amino acids T19A, T116A and K188M, equivalent to the ScASNase1 T64A, T141A and K215A) that also decreased or abrogated the AspG activity^22^. Additionally, the determination of crystallographic structure of the AspG mutants revealed that no significant differences were observed among the mutants and the wild-type enzyme, indicating that mutations do not exert significant effects on the protein structure but only over the enzyme activity^22^. To investigate if the amino acids substitutions also exerted influence over ScASNase1 structure we carried out structural approaches using circular dichroism spectroscopy. The analysis of secondary structure revealed that all mutant enzymes presented structural content slightly different to the wild type enzyme (Fig. 3C; Table 3), similar to the observed to AspG and similar to other mutants using a different approach^22^.

We also assessed the thermal stability of wild-type ScASNase1 using structure circular dichroism spectroscopy. The thermal profile of ScASNase1 revealed that the enzyme is very thermoresistant, presenting a melting temperature of 68.25 ± 0.21 °C (Fig. 3E), which may be related to the high cysteine content present in the enzyme, which in turn may form structural disulphides (Fig. 3A).

### Cytotoxicity of ScASNase1

The cytotoxic potential of ScASNase1 was analysed *in vitro* in three different cell types: HUVEC, as healthy cells that cover the blood vessel wall and are therefore in contact with circulating drugs; REH, considered resistant to treatment with L-ASNase because it has high *ASNS* levels of expression^23^; and MOLT-4, described as a sensitive L-ASNase lineage. Nevertheless, MOLT-4 evolves resistance six weeks after L-ASNase treatment, through the augmented expression of *ASNS*^24^.

**Figure 4:**
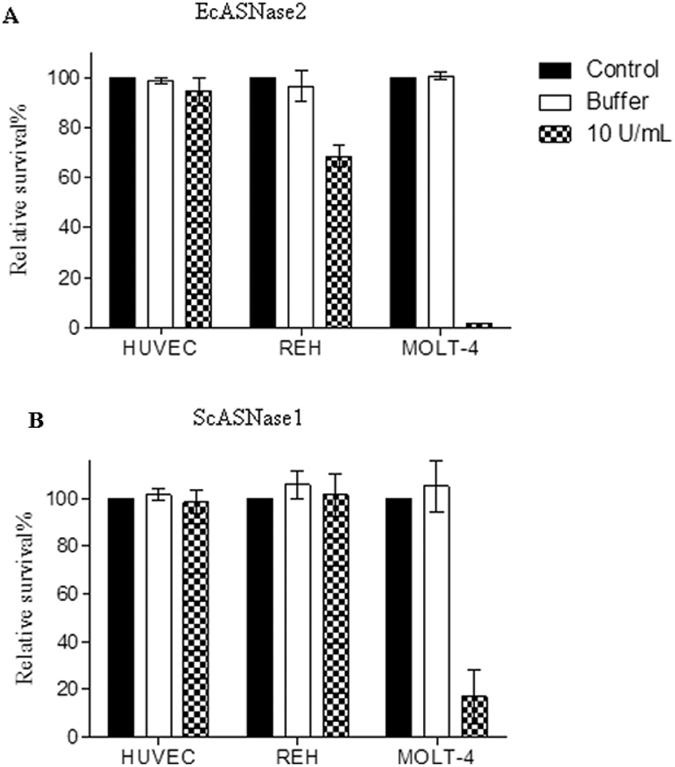
Cytotoxicity assay using HUVEC, REH and MOLT-4 cell lines. (**A**) Relative survival using EcASNase2. (**B**) Relative survival using ScASNase1. Cells were incubated with 10 U/mL of ScASNase1 or EcASNase2 (Prospec–Tany, Israel) for 72 h. The control is represented by the cell culture containing only RPMI 1640 medium with 10% foetal bovine serum. The enzyme buffer was Tris–HCl 50 mM pH 8.8 to ScASNase1 or Tris–HCl 50 mM pH 7.4 to EcASNase2. Error bars were calculated from the mean ± SD (n = 3). Statistical analysis using the ANOVA with Bonferroni test performed using GraphPad Prism 6.05 software showed p < 0.001 versus respective control and buffer.

Cells were incubated with 10 U/mL of ScASNase1 or EcASNase2. Controls were incubated with only RPMI medium or protein buffer for 72 hours (Fig. 4). As expected, the *E. coli* enzyme did not cause toxicity to normal healthy cells HUVEC. However, EcASNase2 treatment killed almost 100% of MOLT-4 cells and 20% of the resistant lineage, REH. Similarly to treatment with EcASNase2, treatment with ScASNase1 did not cause toxicity to normal cells HUVEC and caused death about to 85% of MOLT-4 cells, but REH cells were resistant to ScASNase1 (Fig. 4). Statistical analysis of the data, using ANOVA with the Bonferroni model, demonstrated that the mortality of the REH cells treated with EcASNase2 and MOLT-4 cells in the presence of EcASNase2 or ScASNase1 was significant at a level of p < 0.001, compared with the respective controls.

## Discussion

To our knowledge, we describe here for the first time the characterization of biochemical and antileukemic activity of the ASNase1 from *S. cerevisiae,* providing evidence of its biopharmaceutical potential. Moreover, the heterologous expression of ScASNase1 was obtained with high purity (see supplementary Fig. 1) through one chromatographic step with specific activity of 196.2 U/mg for L-Asn (Fig. 1A). Only Jones and Mortimer^17^ and Dunlop *et al.*^14^ had found specific activity of 0.06 U/mg and 5.4 U/mg, respectively, to ScASNase1^14^,^16^. However, these authors obtained the enzyme by purification of the native protein direct from *S. cerevisiae* through multiple chromatographic steps. The specific activity found in our study is similar to that reported for type II L-ASNase from *E. coli* (EcASNase2), as confirmed here using commercial enzyme as control (223 ± 7 U/mg) (see supplementary Fig. S3).

EcASNase2 and EwASNase2 also show specific activity for L-Gln with values corresponding to a range of 2% and 10% of their L-ASNase activities^4^,^5^. According to our results, ScASNase1 presents glutaminase activity that is 0.38% of L-ASNase activity (Fig. 1B), which is approximately twenty-five times smaller than that of the commercial enzyme EcASNase2 tested in our laboratory (∼10%) (see supplementary Fig. S3).

Glutaminase activity has been demonstrated to be important for effective treatment in ASNS-positive leukemic lines (which express asparagine synthetase), while for the ASNS-negative line (which does not express asparagine synthetase), only L-ASNase activity would be required^25^. However, another study showed that glutaminase activity in ASNS-negative lines is important to increase the death of leukemic cells, and this effect can be associated with the expression of ASNS by subclones, or with the achievement of L-Asn from other cells^26^. According to Anishkin *et al.*^19^, L-Gln is responsible for ensuring the asparaginase activity of the enzyme at submicromolar concentrations of L-Asn, concentrations that are reached in the serum during ALL treatment. L-Gln was a positive modulator, because in these very low concentrations, EcASNase2 would be unable to recognize the substrate, increasing the affinity of the enzyme for L-Asn^19^. This mechanism of regulation justifies the need for L-glutaminase activity for the effective treatment ASNS-negative cells. Nevertheless, this activity is responsible for enhancing adverse effects such as neurotoxicity and hyperammonemia^4^,^11^. Thus, an enzyme with low glutaminase activity and dependence, such as ScASNase1, may be of utility in reducing adverse effects in the treatment of ALL in an ASNS-negative patient.

Dunlop *et al.*^14^ reported a *K*_m_ of 740 μM and suggested that ScASNase1 could not deplete low levels of L-Asn. We demonstrated allosteric behaviour (Fig. 1E,F) with a *K*_*0.5*_ of 75 μM and positive cooperativity, as indicated by n_H_ of 2.2 (Table 1). Allosteric and cytoplasmic L-ASNases are classified as bacterial type I enzymes, such as L-ASNase1 from *E. coli* (EcASNase1), which has n_H_ of 3.5 and *K*_*0.5*_ = 0.4 mM^27^,^28^, and human L-ASNase1 (HsASNase1), which has n_H_ of 3.9 and *K*_*0.5*_ = 11.5 mM^29^. In the same way as for type I enzymes, ScASNase1 is regulated by the substrate to catalyse the hydrolysis of L-Asn but shows high affinity for the substrate and no inhibition by product (Fig. 2C). However, the catalytic efficiency of ScASNase1 is affected by allosteric behaviour and Hill coefficient^30^,^31^. While michaelian EcASNase2 has an efficiency dictated by k_cat_/K_m_ of 10^6^ M^−1^ s^−1^, ScASNase1 efficiency is defined by k_cat_/[K_0.5_]^nH^ reaching a value of 10^4^. This is two orders of magnitude lower than EcASNase2, but it is seven orders higher comparing with EcASNase1 (see Table 1).

Because of allosteric behaviour, the effect of ions was tested. The presence of Mg^2+^ did not change the activity and Zn^2+^ and Ca^2+^ decreased the activity (Table 2); this result is indicative that ScASNase1 is not a metalloprotein^29^. Furthermore, the enzymatic inhibition caused by metal ions and the presence of 10 cysteines in ScASNase1 (Fig. 3A) suggest that certain vicinal sulfhydryl groups are important for protein stabilization, a property described for other L-ASNases^32^,^33^. It is noteworthy to cite that the incubation of EcASNase2 with human serum did not affect the activity, while ScASNase1 presented a significant augment of specific activity (Fig. 2D), suggesting that other serum factors can contribute to positive allosteric regulation of the enzyme.

EcASNase2 and EwASNase2 have high affinity for the substrate, with *K*_m_ in the μM range^34^,^35^,^36^,^37^. Even with a michaelian profile, EcASNase2 has a degree of cooperativity at submicromolar concentrations of L-Asn, which is modulated by L-Gln^19^. The addition of L-Gln during the reaction did not affect the affinity for the substrate or the degree of cooperativity for ScASNase1 (Table 1 and Fig. 2A,B), which is different from that recently described for EcASNase2 and reveals distinct functional characteristics of the yeast enzyme.

Despite structural differences, type I and type II L-ASNases present similarity in their amino acid sequences, and their functional active site residues are conserved throughout evolution^1^,^16^,^38^. The mechanism of action proposed for L-ASNases relies on EcASNase2 observations, in which a first nucleophilic attack of T12 on the substrate is followed by the release of ammonia and formation of the acyl-enzyme complex. In the subsequent step, with K162 and T89, this intermediate undergoes a second nucleophilic attack by a water molecule, resulting in hydrolysis of the acyl-enzyme intermediate, producing the aspartic acid, leaving the free enzyme^39^. The hydrogen bonds formed between Y25 and T12 promote the correct orientation of T12 for catalysis^40^. A comparison of the primary structure of EcASNase2 and EwASNase2 with ScASNase1 revealed that the active site residues involved in catalysis are conserved in ScASNase1 (Fig. 3A,B). Single substitutions of the T64, Y78, T141 and K215 residues to alanine revealed that all mutants have significant loss of activity compared to the wild-type enzyme (Fig. 3C). Structural analysis of secondary structure content revealed that all mutant enzymes presented similar α-helix and β-sheet content relative to the wild-type ScASNase1 indicating that the amino acids substitutions performed in this work did not affect the integrity of the enzyme structure (Table 3).

Additional analyses aiming to evaluate enzyme thermostability showed that ScASNase1 possesses high thermostability (∼68 °C – Fig. 3E), in agreement with kinetic thermal analysis, which revealed that the enzyme retains >60% of its asparaginase activity at 50 °C (Fig. 1D).

The main characteristic of bacterial type II enzymes is their antineoplastic potential. The first characterization of ScASNase1 demonstrated low specific activity and a *K*_m_ of 740 μM for L-Asn^14^; this affinity would not allow the depletion of human serum levels of L-Asn, since an enzyme has to be very active at low substrate concentrations to present antineoplastic potential^36^. Yun *et al.*^27^ demonstrated the allosteric profile of EcASNase1 and proposed that L-ASNases type I cannot be considered for therapeutics because it does not deplete low levels of L-Asn^27^. Considering that, asparagine concentration in circulating human serum is 50 μM^41^, using the equation 1, equal amount of enzymes and kinetic values of Table 1, we can compare the velocity of reactions in physiological conditions among EcASNase1, EcASNase2 and ScASNase1.


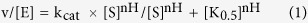


Equation 1: where v is initial velocity of reaction; [E] is total enzyme concentration; k_cat_ is the turnover constant; [S] is the substrate concentration; K_0.5_ is the concentration of substrate in which enzyme reaches half of maximal velocity and n_H_ is the Hill coefficient^31^.

In this prediction, EcASNase2 exerts its antileukemic activity with v = 92 μM/s; ScASNase1 would present v = 63 μM/s, while EcASNase1 would show v = 0.005 μM/s. This strongly suggests that, at least in catalytic kinetic terms, ScASNase1 potentially exerts asparaginase activity compatible with antileukemic effect.

In 2013, a study with encapsulated ScASNase1 showed that this enzyme can induce the death of leukemic cell lines *in vitro*^42^. Here, we corroborated this data and also showed that ScASNase1 is active in physiological conditions (pH 7.4 at 37 °C), with optimum activity even at pH 8.6 and 40 °C (Fig. 1C,D) and kills 85% of human acute lymphoblastic leukaemia MOLT-4 cells *in vitro* (Fig. 4). MOLT-4 is an important cell line that has been used to investigate the effect of other L-ASNases, such as L-ASNase from *Erwinia carotovora* and *Helicobacter pylori.* In these cases, 10 U/mL of *Erwinia carotovora* or *Helicobacter pylori* caused mortality of 83% and 30%, respectively, in MOLT-4 cells^43^,^44^. Using the same concentration of EcASNase2, the mortality of MOLT-4 cells was 95%^43^. The REH cell line uses L-Gln pathway asparagine synthetase for the synthesis of L-Asn^23^. The low glutaminase activity of ScASNase1 prevents the mortality of this lineage. However, the antileukemic potential of ScASNase1 demonstrated here was superior to that observed for type II enzymes from *Erwinia carotovora* and *Helicobacter pylori* and is equivalent to that caused by EcASNase2 in MOLT-4 cells. It is noteworthy that the non-bacterial origin of ScASNase1 may present an additional advantage in the antileukemic treatment of sensitive lines, reducing the adverse effects due to low glutaminase activity.

In conclusion, our results show that ScASNase1 has specific activity of 196.2 U/mg, which is compatible with the enzymes used in the treatment of ALL and almost forty-fold higher than previously characterized. In addition, it has allosteric behaviour with a *K*_0.5_ of 75 μM, showing a high affinity for the substrate L-Asn. Residues T64-Y78-T141-K215 play important roles in enzyme catalysis. We suggest that ScASNase1 may have interesting antineoplastic properties, because ScASNase1 shows toxicity for leukemic cells but not to normal healthy cells (HUVEC), can reduce adverse effects due to low glutaminase activity and may be less immunogenic than bacterial enzymes. ScASNase1 is classified as an enzyme belonging to bacterial type II, has high affinity for the substrate and exhibits antineoplastic properties. However, ScASNase1 also presents characteristics of bacterial type I enzyme, such as allosteric behaviour and cytoplasmic localization in yeast. Additional structural and antileukemic studies that are being conducted by our research group could help to elucidate the active site microenvironment and unique properties of ScASNase1.

## Methods

### Gene cloning, protein expression and purification of ScASNase1

The *ASP1* gene was isolated from genomic DNA from the BY4741 strain of *S. cerevisiae* by polymerase chain reaction, using the oligonucleotides SC_Asp1F 5′ GGGAAATTCCATATGTTACCAAGAATCAAAATCTTGGG 3′ and SC_Asp1R 5′ CGCGGATCCTCACCACCATAGAC 3′ with restriction site adaptors to *Nde* I and *Bam* HI (Exxtend Biotecnologia São Paulo, Brazil). The PCR product was cloned into *Nde* I and *Bam* HI restriction sites of the pET15b vector (Novagen – Merck Millipore). pET15b-*ASP1* was used to transform *E. coli* DH5α. Single colonies were selected; their plasmids were extracted and sequenced using the BigDye Terminator v3.1 Cycle Sequencing Kit using the ABI 3730 DNA Analyser (Thermo Scientific) automatic sequencer to confirm gene integrity. The correct constructions were used to transform *E. coli* BL21 (DE3) (Novagen – Merck Millipore) strain by electroporation.

Transformed *E. coli* BL21 (DE3) containing pET15b-*ASP1* were grown overnight at 37 °C in 100 mL of medium LB (10% tryptone, 5% NaCl and 5% yeast extract) containing 50 μg/mL of carbenicillin and then transferred to 1 L of fresh LB containing carbenicillin (50 μg/mL) and grown to OD_600nm_= 0.6–0.8. Protein expression was induced by IPTG addition to a final concentration of 1 mM for 3 hours at 37 °C. The cells were harvested by centrifugation at 4.000 × *g*/4 °C/20 min, and the pellet was suspended with start buffer (20 mM sodium phosphate pH 7.4; 300 mM NaCl; 20 mM imidazole) and added to PMSF 1 mM. Cell disruptions were performed by sonication with 30% amplitude, sonicating for 24 cycles of 5 seconds and resting for 15 seconds in ice. The cellular lysate was treated with 1% sulfate streptomycin in ice for 20 min. The suspension was clarified by centrifugation at 16.000 × *g*/4 °C/30 min and homogenised by filtration using a 45-μm membrane (Merck–Millipore) and applied to a Hi-Trap nickel-affinity column (GE Healthcare) for purification. The column was equilibrated with 10 volumes of start buffer (20 mM sodium phosphate pH 7.4; 300 mM NaCl; 20 mM imidazole); the protein extract was applied, and the unbound proteins were washed with 5 column volumes using a stepwise gradient of imidazole (20 mM sodium phosphate pH 7.4; 300 mM NaCl; initializing with 50 mM, followed by 100 mM and 150 mM imidazole); ScASNase1 was eluted with 20 mM sodium phosphate pH 7.4; 300 mM NaCl; 500 mM imidazole.

Protein purification and integrity were evaluated by SDS–PAGE according to the method of Laemmli (1970). The ScASNase1 was applied to a PD-10 Desalting Column (GE Healthcare), using 20 mM Tris–HCl pH 8.8 buffer. The protein concentration was measured by spectrophotometry at λ = 280 nm, with an extinction coefficient of ScASNase1 (ε=25955 M^−1^ cm^−1^) obtained using the ProtParam tool (http://web.expasy.org/protparam/).

### Mutagenesis of ScASNase1

Mutants T64A, Y78A, T141A and K215A were obtained using a QuikChange Site-Directed Mutagenesis Kit (Agilent Technologies) with pET15b-*ASP1* as the template and the following primers: ASP1T64A_F 5′GGGTACCGGTGGTGCG ATTGCATCGAAAGC 3′ and ASP1T64A_R 5′GCTTTCGATGCAATC GCACCACCGGTACCC 3′; ASP1Y78A_F 5′AAACTGCCGGCGCGCATGTTGACCTGACC 3′ and ASP1Y78A_R 5′GGTCAGGTCAACATGCGCGCCGGCAGTTT 3′; ASP1T141A_F 5′ATTACCCATGGGGCCGATACGCTAT 3′ and ASP1T141A_R 5′ ATAGCGTATCGGCCCCATGGGTAAT 3′; ASP1 K215A_F 5′ TCTGGTTACTACATTACTGCCACGAATGCAAATAGTTTGG 3′ and ASP1 K215A_R 5′ CCAAACTATTTGCATTCGTGGCAGTAATGTAGTAACCAGA 3′. The products of the reaction were treated with *Dpn* I to remove the original methylated plasmids. The *E. coli* XL1-Blue strain was used as host in the transformations. The plasmids were sequenced, as described before, to confirm the codon substitutions, and positive ones were used to transform *E. coli* strain BL21 (DE3) (Novagen – Merck Millipore) by electroporation. The recombinant enzymes were expressed and purified as described for the wild-type enzyme.

### Enzyme activity assay

To determine the level of L-ASNase-specific activity, we measured the ammonia produced by the hydrolysis of L-Asn (Sigma–Aldrich/USA) catalysed by the enzyme through Nessler’s reagent (Merck–Millipore). The protocol used was adapted to a microplate reader according to the manufacturer’s instructions. The reaction containing 50 mM Tris–HCl (pH 8.8), 20 mM L-Asn and 36–540 ng of enzyme was incubated at 37 °C for 20 min; the reaction was stopped with trichloroacetic acid (TCA) 1.5 M and then diluted in water (10×) followed by the addition of Nessler’s reagent. The same assay was used to determine the interference of L-Aspartate (L-Asp) in the reaction, using 10 mM L-Asn + 20 mM L-Asp. For the mutant isoforms T64A, Y78A, T141A and K215A, 0.03–0.27 mg protein were used with 20 mM L-asparagine. The reactions were incubated for 1 hour at 37 °C. The results were recorded spectrophotometrically at λ = 440_nm_ using the SpectraMax Microplate Reader (Molecular Devices). The standard curve was constructed with ammonium sulfate using concentrations ranging from 0 to 20 μmol/mL^45^.

Glutaminase activity was estimated spectrophotometrically using an NADH oxidation coupled assay adapted from Balcão, *et al.*^46^. The L-glutamate (L-Glu) produced in the hydrolysis of L-Gln by L-ASNase is used for the synthesis of α-ketoglutarate in the presence of L-glutamate dehydrogenase (GDH) (Sigma–Aldrich/USA) and β-nicotinamide adenine dinucleotide (NADH) (Sigma–Aldrich/USA). The reactions were performed using microplates as follows: 50 mM Tris–HCl pH 8.0, 20 mM L-Gln, 0.13 mM NADH, 0.5 U GDH (diluted in 50 mM sodium phosphate pH 7.4; 50% glycerol) and 0 mg to 0.09 mg of ScASNase1 enzyme or the commercial enzyme EcASNase2 (Prospec–Tany, Israel), which was used as a positive control. The absorbance was measured at λ = 340_nm_ at 37 °C, and the extinction coefficient used was ε = 0.85 μmol^−1^ cm^−1^.

### Effect of pH and temperature on ScASNase1

To determine the optimum pH for ScASNase1, enzyme activity was measured at 37 °C in different buffers: acetate pH 4.0; sodium phosphate pH 6.0, 7.0 and 12.0; Tris–HCl pH 8.0 and 9.0; sodium bicarbonate pH 10.0. To determine the optimum temperature, enzyme activity was measured from 25 °C to 65 °C. After 20 min of reaction, the ammonium generated was measured by Nessler’s reagent as described above.

### Determination of specific activity of ScASNase1 in the presence of Zn^2+^, K^+^, Mg^2+^, Ca^2+^ and human serum

The final concentrations of reagents were used in all assays: L-asparaginase (1–30 nM), Tris–HCl buffer pH 8.8 50 mM, 20 mM L-asparagine. The enzyme was pre-incubated at 37 °C for 30 minutes with each compound at concentrations of 0.5 or 10 mM from Zn^2+^, K^+^, Mg^2+^ or Ca^2+^. To assay with human serum, ScASNase1 and EcASNase2 were pre-incubated at 37 °C for 60 minutes with 10% human serum (Agilent Technologies).

The reactions were incubated at 37 °C for 20 minutes with L-asparagine. The ammonium release was measured using Nessler’s reagent as described above.

### Kinetic analysis

The kinetic parameters of purified ScASNase1 to hydrolysis of L-Asn were determined by spectrophotometry through NADH-consumption-coupled assay as adapted from Balcão, *et al.*^46^. The ammonia released is used in the production of L-Glu in the presence of GDH and reduced β-nicotinamide adenine dinucleotide (β-NADH) (Sigma). NADH oxidation was measured spectrophotometrically at λ = 340_nm_ at 37 °C, and the extinction coefficient used was ε = 0.85 μmol^−1^ cm^−1^. Microplates received 50 mM Tris–HCl pH 8.0; 0.07 mM, 0.1 mM, 0.3 mM, 0.5 mM, 0.7 mM, 1.5 mM, 2.0 mM and 2.5 mM of L-Asn, 0.13 mM β-NADH, 1 mM α-ketoglutarate 0.5 U GDH (diluted in 50 mM sodium phosphate pH 7.4; 50% glycerol) and 180 ng of ScASNase1. A second assay was conducted at the same concentrations of L-Asn and ScASNase1 with added L-Gln at a ratio of 1:16 for each concentration of L-Asn. The concentrations of L-Gln used were 1.12 mM, 1.6 mM, 4.8 mM, 8.0 mM, 11.2 mM, 24 mM, 32 mM and 40 mM. The substrate affinity and turnover number were determined using non-linear regression analysis of experimental steady-state data using GraphPad Prism version 6.05 software. Kinetic parameters were determined under the same conditions described above for commercial enzyme EcASNase2 (Prospec–Tany, Israel).

### Circular dichroism spectroscopy of ScASNase1

The CD spectra of ScASNase1 native and reduced proteins were obtained using a 0.1 cm path length cuvette containing 10 μM of protein sample in 5 mM Tris buffer (pH 7.4). The assays were carried out at 25 °C in a Jasco J-815 spectropolarimeter (Jasco Inc.). The spectra are representative of an average of eight scans recorded from 190 to 260 nm. The content of secondary structures in each protein (wild-type and mutants) was estimated using CDNN 2.1 software^47^. Melting profiles at a constant wavelength of 222 nm, θ_222_, were recorded while the sample was heated from 20 to 95 °C, with a 1 °C increment/min.

### Cells culture and cytotoxicity assay

The leukaemia human cell lineage MOLT-4 and REH, as well as the Human Umbilical Vein Endothelial Cells (HUVEC) were obtained from the Banco de Células do Rio de Janeiro (RJ/Brazil). Cells were maintained in RPMI 1640 medium with 10% foetal bovine serum (v/v) and incubated at 37 °C in a 5% CO_2_ incubator. These procedures followed the ATCC instructions (http://www.atcc.org/). After reaching confluence, cell lineages were centrifuged at 600 × *g* at 4 °C for 10 min and suspended in fresh RPMI medium. MOLT-4, REH or HUVEC, at 1.0 × 10^4^ cells/well, were incubated in 24-well microplates with and without 10.0 U/mL of ScASNase1 or EcASNase2. As a control, the cells were incubated with RPMI medium and with protein buffer (50 mM Tris–HCl pH 8.8 for ScASNase1 and 50 mM Tris–HCl pH 7.4 for EcASNase2) for 72 hours at 37 °C in a 5% CO_2_ incubator. Cells were centrifuged at 600 × *g* at 4 °C for 10 min, then stained with Trypan blue (Sigma–Aldrich/USA) to select dead cells. Cells were counted in a Neubauer chamber to determine cell viability, which is expressed as a percentage of living cells.

### Statistical Analysis

All analyses were performed, at least, in triplicate. Results were represented as mean ± standard deviation (SD). The statistical analysis was performed using ANOVA, considering p ≤ 0.05, and using GraphPad Prism version 6.05 software.

## Additional Information

**How to cite this article**: Costa, I. M. *et al.* Recombinant L-asparaginase 1 from *Saccharomyces cerevisiae*: an allosteric enzyme with antineoplastic activity. *Sci. Rep.* **6**, 36239; doi: 10.1038/srep36239 (2016).

**Publisher’s note:** Springer Nature remains neutral with regard to jurisdictional claims in published maps and institutional affiliations.

## Supplementary Material

Supplementary Information

## Figures and Tables

**Table 1:** Kinetic parameters of ScASNase1 to hydrolyse of L-asparagine and comparison with EcASNases.

EcASNase2	*K*_m_ (μM)	*V*_*max*_ (μmol/min)	*k*_cat_ (s^−1^)	*k*_cat_/*K*_m_ (M^−1^.s^−1^)	*n*_*H*_
L-asparagine	23 ± 5	0.032 ± 0.00061	134 ± 2	5.8 ×10^6^	1.1 ± 0.1
ScASNase1	*K*_*0.5*_(μM)	*V*_*max*_ (μmol/min)	*k*_cat_ (s^−1^)	*k*_cat_/[*K*_*0,5*_] ^*n*H^ (M^−1^. s^−1^)	*n*_H_
L-asparagine	75 ± 27	0.042 ± 0.0011	217 ± 14	1.6 × 10^4^	2.2 ± 0.3
L-asparagine +L-glutamine	122 ± 31	0.097 ± 0.0023	523 ± 34	1.3 × 10^4^	2.2 ± 0.2
EcASNase1a*					
L-asparagine	400 ± 50	−	7.4 ± 0.3	5.8 × 10^−3^	3.5 ± 0.3

^a*^Kinetics data from Karamitros and Konrad, 2014 – using the same NAD+ coupled assay^29^.

**Table 2:** Relative activity of ScASNase1 in the presence of different effectors.

Effector	Concentration	Relative activity %
K^+^	5.0 mM	109.7%
Mg^2+^	10.0 mM	100.5%
Zn^2+^	0.5 mM	7.3%
Ca^2+^	0.5 mM	0%

**Table 3:** Secondary structure analyzed by circular dichroism spectroscopy.

	ScASNase1	T64A	Y78A	T141A	K215A
Helix	16.50%	16.36%	16.12%	14.30%	16.93%
Antiparallel	26.65%	26.10%	26.82%	28.21%	26.21%
Parallel	9.25%	9.37%	9.31%	9.60%	9.22%
Beta-turn	16.32%	16.26%	16.23%	15.67%	16.44%
Random Coil	31.28%	31.91%	31.48%	32.22%	31.22%

The secondary structure contents were obtained using CDNN software.
