# Supplementary material for: Recombinant L-asparaginase 1 from Saccharomyces cerevisiae: an allosteric enzyme with antineoplastic activity
Source: Sci Rep. 2016 Nov 8;6:36239. doi: 10.1038/srep36239 (PMC5099943; doi:10.1038/srep36239)
Supplement: Supplementary Information [file srep36239-s1.doc]

Supplemental Material

Recombinant L-asparaginase 1 from *Saccharomyces cerevisiae*: an allosteric enzyme with antineoplastic activity

Iris Munhoz Costa^1^, Leonardo Schultz da Silva^2^, Beatriz de Araujo Bianchi Pedra^1^, Mariana Silva Moreira Leite^1^, Sandra H. P. Farsky^3^, Marcos Antônio Oliveira^2^, Adalberto Pessoa^1^, and Gisele Monteiro^1^


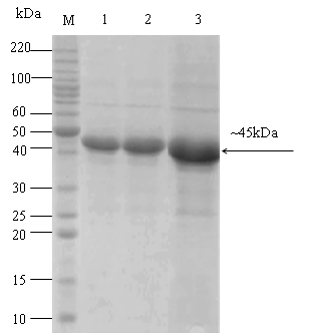


**Figure S1.** SDS–PAGE (14% gel) of recombinant ScASNase1 enzyme purification. The gel was stained with Coomassie Brilliant Blue R-250. Line M: molecular mass marker; line 1–3: recombinant ScASNase1 enzyme obtained after purification by IMAC.

**Figure S2.** Specific activity ScASNase1 for L-Asn: Plot of the reaction velocities (*V_0_*) of L-Asn hydrolysis as a function of mg of purified ScASNase1 as measured by coupled assay with NADH oxidation.

**Figure S3 -** Assay with commercial EcASNase2 (Prospec–Tany, Israel). **A***,* Specific activity EcASNase2 for L-Asn: Plot of the reaction velocities (*V_0_*) of L-Asn hydrolysis as a function of mg of protein as measured by Nessler’s reagent. **B***,* Specific activity of ECASNase2 for L-Asn: Plot of the reaction velocities (*V_0_*) of L-Asn hydrolysis as a function of mg of protein measured by coupled assay with NADH oxidation. **C***,* Specific activity for L-Gln as measured by coupled assay with NADH oxidation: plot of the reaction velocities (*V_0_*) of L-Gln hydrolysis as a function of mg of protein. **D***,* EcASNase2 kinetics, activity dependence on substrate concentration plot. **E***,* the Lineweaver–Burk plot of data. Points represent means ± SD (n = 3).


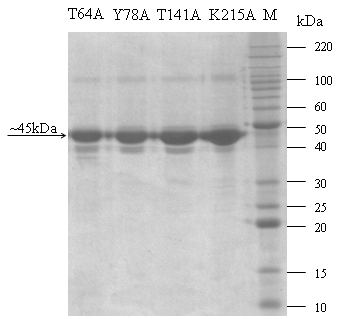


**Figure S4**. SDS–PAGE (14% gel) of recombinant ScASNase1 mutant enzyme purification. The gel was stained with Coomassie Brilliant Blue R-250. M: molecular weight marker; T64A, Y78A, T141A and K215A ScASNase1 mutant enzymes were obtained after purification by IMAC.
